# Pilot study: a descriptive-retrospective analysis of SARS-CoV-2 variants distribution and phylogenesis in the Phlegraean area

**DOI:** 10.3389/fmolb.2025.1536953

**Published:** 2025-02-27

**Authors:** Maria Cristina Mazzarella, Stefano Cristiano, Dilia Rea, Nicola Mazzarella, Martina Addeo, Silvia Iannelli, Geppino Falco, Mariarita Brancaccio, Tiziana Angrisano

**Affiliations:** ^1^ ISM Baia, Genetic Analysis Laboratory, Naples, Italy; ^2^ Department of Advanced Biomedical Sciences, University of Naples Federico II, Naples, Italy; ^3^ Department of Biology, University of Naples Federico II, Naples, Italy; ^4^ Department of Public Health, University of Naples Federico II, Naples, Italy

**Keywords:** pilot study, SARS-CoV-2, genetic variants, next-generation sequencing, virus evolution

## Abstract

COVID-19 disease, caused by SARS-CoV-2 virus, marked the pandemic era, opening the way to next-generation sequencing in the viral diagnostic field. SARS-CoV-2 viral genome sequencing makes it possible to identify mutations in the virus and to track the diffusion of these variants in specific geographic area and in time. Variant sequences help understand how the virus spreads and how it can be contained, as well as for developing more effective vaccines and therapies. Indeed, monitoring the evolution of a virus allows us to quickly detect the potential selection of a super mutation, which can make a virus even more contagious and dangerous in terms of human health consequences. In light of this, in our pilot study, we decided to profile the SARS-CoV-2 genome, recruiting 38 patients divided according to age, sex, vaccination status and symptoms, ascertaining their positivity to the virus. Specific strains of SARS-CoV-2 have been identified and effective through next-generation sequencing. This analysis made it possible to obtain information on the variants of the virus and their spread in the Campania region of the Phlegraean area, in the municipalities of Bacoli, Pozzuoli and Monte di Procida from December 2021 to February 2023 and on the effect of long-term measures COVID-19 in our sample. The advantage of using NGS in diagnosis is the introduction of tests on many genes in a relatively short time and at relatively low costs, with a consequent increase in a precise molecular diagnosis and helps to identify *ad personam* therapies.

## 1 Introduction

Severe Acute Respiratory Syndrome CoronaVirus 2 (SARS-CoV-2), since its emergence in 2019, has caused more than 765 million cases of COVID-19 and more than 6.9 million deaths as of December 2022 (WHO, 2022) (World Health Organization). On 5 May 2020, the WHO officially declared the global COVID-19 epidemic and pandemic (Hui et al., 2020). COVID-19 causes flu-like clinical symptoms such as fever, headache, cough, sore throat, breathing difficulties and dysentery (Li et al., 2020; Scudiero et al., 2021; Brancaccio et al., 2022; Brancaccio et al., 2021). SARS-CoV-2 is a positive-sense single-stranded RNA (+ssRNA) virus that, despite the presence of an Exonuclease proof-reading activity (Exonuclease activity, NSP14), cannot ensure the integrity and persistence of the genome, determining elevated mutation rates in its genome. This imperfect mechanism enables the natural selection of advantageous traits, such as increased virulence, adaptability, and progression (Faraji et al., 2024). Host infection and thus the life cycle of SARS-CoV-2 begins with the Spike (S) protein, a type I membrane glycoprotein embedded in the envelope of the virus with the most variable sequences among the coronavirus genomes (Woo et al., 2010), which binds the receptor angiotensin-converting enzyme 2 (ACE2) on the target cell. Specifically, the efficiency of virus entry into the host cell depends on the cleavage of the S1/S2 site of the Spike protein mediated by the surface transmembrane protease Serine 2 (TMPRSS2) and/or endolysosomal cathepsin L. This interaction mediates the fusion of the virus membrane with the endosomal compartments of the target cell (Khan et al., 2021). To date, COVID-19 represents a pandemic model of infection whose severity and variability of symptoms have brought the world community to its knees, prompting all scientists to study its genome and evolution as best they can to understand its response in the patient (Vakil and Trappe, 2022). In this regard, in 2008 a repository was created for sharing all influenza virus genetic sequences and metadata downstream of the HIN1 epidemic, the Global Initiative on Sharing Avian Influenza Data (GISAID) (Shu and McCauley, 2017). Since 2020, more than 15.700.000 SARS-CoV-2 sequences have been entered into the GISAID database, each different from the other (https://gisaid.org/). In December 2019, SARS-CoV-2 was first identified in pneumonia cases in Wuhan, China” (Wu et al., 2020; Zhou et al., 2020); the first massive sequencing data collected from epidemiological screenings started in the last months of 2020 (Oude Munnink et al., 2021). Therefore, a new classification introduced by the WHO has identified several SARS-CoV-2 variants documented during the pandemic, and among these, some have been identified as variants of concern (VOCs) with a public health impact (Marianna et al., 2022). In addition to the main variants VOCs, there were also other intermediate variants transitioning between the main ones, which, however, although not identified as relevant because they do not have a particular impact on public health, are also present within the complete classification. Through the Open Source Nextstrain project, it is possible to continuously update the publicly available data in GISAID on the pathogen genome to aid epidemiological understanding and improve the scientific response to the pandemic (Flores-Vega et al., 2022). Environmental, demographic, and clinical factors also impact the severity of COVID-19 influence (Ferreira et al., 2022). Therefore, we aimed to accomplish a picture in space and during the evolution of the SARS-CoV-2 variant in a specific area of the Campania region.

Our observational pilot study aimed to analyze SARS-CoV-2 strains that spread specifically in the Phlegraean area. Patients were recruited between December 2021 and February 2023, which showed us the prevalent variants that caused the disease in that specific geographical area. We identified 15 Omicron variants through next-generation sequencing (NGS), which were grouped through phylogenetic analysis to trace the relationship between the first Omicron variant (B.1.1.529) and the original SARS-CoV-2 sequence.

We also classified nucleotide mutations in the spike protein, the region with the highest mutation rate and a crucial target for vaccine design. Finally, to investigate potential long-term effects associated with specific variants, patients were contacted 1–3 years post-infection. The need for advanced genomic tools, such as NGS, in molecular diagnostics represents a new frontier for personalized medicine.

## 2 Materials and methods

### 2.1 Samples collection

The samples were collected in accordance with the Bioethics Committee of the University of Naples Federico II, Ref: 72/2024 and stored at −80°C. The patients included in the study were between 18 and 80 years old and had achieved at least two vaccine doses. A total of 38 nasopharyngeal swab samples were selected from SARS-CoV-2 positive samples (from December 2021 to February 2023) in the Phlegraean area, particularly in the municipalities of Bacoli, Pozzuoli, and Monte di Procida, at the ISM BAIA analysis laboratory of Naples. In Supplementary Table S2, the patient’s symptoms were indicated. Testing was performed by the qRT-PCR method through a quantitative reverse transcriptase-polymerase chain reaction assay as indicated by the MOLgen SARS-CoV kit protocol −2 Real-time RT-PCR Kits (Adaltis S. r.l, Roma, Italy). The nasopharyngeal samples were collected in a 5-mL tube containing 2 mL of sample deactivating buffer (Thermo Fisher Scientific, Waltman, MA) composed of 0.9% of normal saline raisin. The sample selection criterion for whole-genome sequencing was based on high viral load predicted by the low cycle threshold (Ct) values of ≤25 on qRT-PCR analysis.

### 2.2 RNA extraction and cDNA synthesis

The viral RNA was extracted from nasopharyngeal swabs using the Viral RNA/Viral Nucleic Acid Mini kit (Thermo Fisher Scientific) following the manufacturer’s instructions. Then, the viral RNA was quantified using kit Qubit RNA IQ Assay (TermoFisher Scientific, Massachusetts, United States) following the manufacturer’s instructions. From sample preparation to loading into sequencing flow cells, the protocol indicated in the Illumina CovidSeq Ruo kit was per-formed.

The complementary cDNA library was constructed using Illumina CovidSeq Assey kit system (Illumina Covidseq, San Diego, CA, United States) compatible with iSeq 100 sequencing, following the manufacturer’s instructions. Briefly, 8.5 µL of RNA was retrotranscripted with First Strand cDNA Master Mix and then the strand was amplified by COVIDSeq PCR Master Mix (Illumina, San Diego CA, United States). The reactions were incubated for 5 min at 25°C, then 10 min at 50°C for reverse transcription and enzyme activation, and 5 min at 80°C. These steps were carried out in a thermocycler RotorGene-Q (QIAGEN, Hilden, Germany).

### 2.3 Library preparation and data processing

Library preparation and high throughput genomic sequencing were performed at the ISM Baia laboratory in Pozzuoli, Italy, and carried out according to the Illumina CovidSeq Ruo kits available on the Illumina website. After incubation at room temperature, cDNAs were vortexed at 1,600 rpm for 1 min and centrifugated at 1,000 g for 1 min.The cDNA library was fragmentated and tagged with Tagmentation Master Mix. The pooled amplified fragments undergo tagmentation and a second round of PCR amplification using a PCR master mix and unique index adapters. After amplification, indexed libraries are pooled and cleaned using purification beads. The libraries were quantified with the Qubit dsDNA HS Assay kit (TermoFisher Scientific, Massachusetts, United States), normalised to 1 nM and then brought to a final concentration of 75 pM. Finally, 20 µL was loaded onto the flow cell and sequenced on an Illumina iSeq 100 System (Illumina Inc., San Diego, CA, United States) to produce 2 × 150 bp paired-end reads in a BCL (o FastQ) format. The BCL files were automatically converted to fastQ format by the Local Run Manager software (v3) after quality check and normalization. Sequence analysis and lineage identification were performed on the BaseSpace Sequence Hub platform with the Dragen COVID Lineage application (v3.5.7) that uses the Pangolin (Phylogenetic Assignment of Named Global) algorithm to conduct lineage and clade analysis. The final output CSV files contain information on the type of variant and sub-variant found, the coverage of the analysis, and the methods of analysis used by the app on the genomic and amino acid mutations found for each sample. All technical information about Base Space Hub and Dranger COVID lineage is available on the Illumina website. In contrast, more detailed information on the Pangolin algorithm and its components can be accessed in the various repositories in the CoV-Lineages area of GitHub (Sadasivan et al., 2023; Jackson, 2022; Turakhia et al., 2021). SARS-CoV-2 raw sequences from the Phlegraean area have been deposited in the GISAID database (https://gisaid.org/) under the accession number indicated (Supplementary Table S1).

### 2.4 Phylogenetic analysis

UPGMA (Unweighted Pair Group Method with Arithmetic mean) hierarchical clustering method was used to infer a phylogenetic tree by iteratively clustering sequences based on their pairwise distances (Sneath PHAaS, 1973). The percentage of replicate trees where the associated taxa clustered together was assessed by a bootstrap test with 500 replicates (Felsenstein, 1985). Evolutionary distances were determined by the Maximum Composite Likelihood method (Tamura et al., 2004) and expressed as the number of base substitutions per site. Ambiguous codon positions were filtered out for each pair of sequences through pairwise deletion. The evolutionary analyses were performed using MEGA11 (Molecular Evolutionary Genetics Analysis Version 11) software (Tamura et al., 2021).

### 2.5 Mutational analysis

Individual variants sequences were analyzed to investigate the genetic mutations resulting in amino acid alterations within the Spike protein. A thorough comparison was undertaken between the mutations present in the Spike protein of each of the 15 variants identified in the sequencing phase and those characterizing the structure of the Spike protein of Omicron B.1.1.529. Then, the focus was specifically directed towards the mutations associated with the patients that exhibited heightened and comprehensive symptoms during the patient’s medical history assessment. The threshold of significant frequency of mutation was fixed at 25%.

### 2.6 Data analysis

All data are analysed and correlated using the GraphPad software.

## 3 Results

### 3.1 Characterization of omicron variants in patient samples: frequency and symptom associations

Detailed clinical features of patients are listed in Table 1. Briefly, a total of 38 patients were collected from three sites: Bacoli (n = 12), Monte di Procida (n = 11) and Pozzuoli (n = 15) (Table 1). Samples were stratified by sex, age and symptom severity grade. Based on mutation profiles identified by Nextclade analysis, sequences have been categorized into five primary Pango lineages of Omicron (or clades): 22A, 22B, 22E, 21K, and 21L. Among these clades, 21K and 22B were the most prevalent (12 patients), followed by 21L (9 patients) and then 22E (4 patients) and 22A (1 patient) (see Table 2). Within these categories, 15 variants have been identified (Table 2).

**TABLE 1:** Characteristics of patients positive for SARS-CoV-2 (N = 38).

Variables	Modality	N. of patients
Sex	Female	20
	Male	18
Age (year)	18–25	2
	30–40	11
	41–50	7
	51–60	4
	61–70	7
	>70	7
Urban community	Bacoli	12
	Monte di Procida	11
	Pozzuoli	15
Symptoms severity grade	Asymptomatic	4
	Mild	13
	Severe^a^	21

^a^ Associated with respiratory difficulties.

**TABLE 2:** Distribution of SARS-CoV-2 Pango lineages variants, clades and variants among our sequence concerning sex of patients.

Clade	N. patients	Variants	Males	Females
21K	12	BA.1	4	2
		BA.1.1	2	0
		BA.1.21.1	0	1
		BA.1.17.2	1	2
21L	9	BA. 2	3	4
		BA. 2.9	1	0
		BA. 2.10.1	1	0
22A	1	BA. 4	1	0
22B	12	BA. 5.1	1	4
		BA. 5.1.10	1	0
		BA. 5.2	1	3
		BA. 5.2.3	1	0
		BE.1	1	0
22E	4	BQ.1.1	1	2
		BQ.1.23	2	1

Our data reveal that 12 patients were infected with the Omicron 21K clade, while another 12 had the 22B clade.

Furthermore, our investigation into the distribution of Omicron variants by sex uncovered interesting differences: male patients were affected by every variant except for BQ.1.23 and BA.1.21.1, whereas female patients were impacted by only 8 out of the 15 variants identified (Table 2).

**FIGURE 1:**
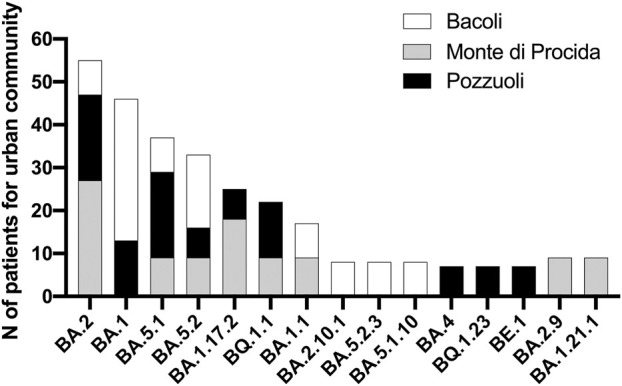
Analysis of variant distribution in urban community. Sequencing data were correlated with sampling in the Phlegraean area: Bacoli, Monte di Procida and Pozzuoli and analyzed by GraphPad software.

Each patient filled out a questionnaire regarding their symptom history (Table 1), and the collected data were processed and organized based on the variants identified during sequencing. Notably, the variants associated with the highest frequency of symptoms were BA.2 (n = 7; 18%), BA.5.1 (n = 5; 13%), and BA.5.2 (n = 4; 10.5%). These variants also represented the largest percentages within our Phlegraean patient cohort (Figure 1). Specifically, the BA.2 and BA.5.1 variants accounted for 20% in Pozzuoli. Also, BA.2 reached 27% in Monte di Procida, while BA.1 was the most common variant in Bacoli (33%) (Figure 1).

### 3.2 Phylogenetic analysis of omicron clades and variants

**FIGURE 2:**
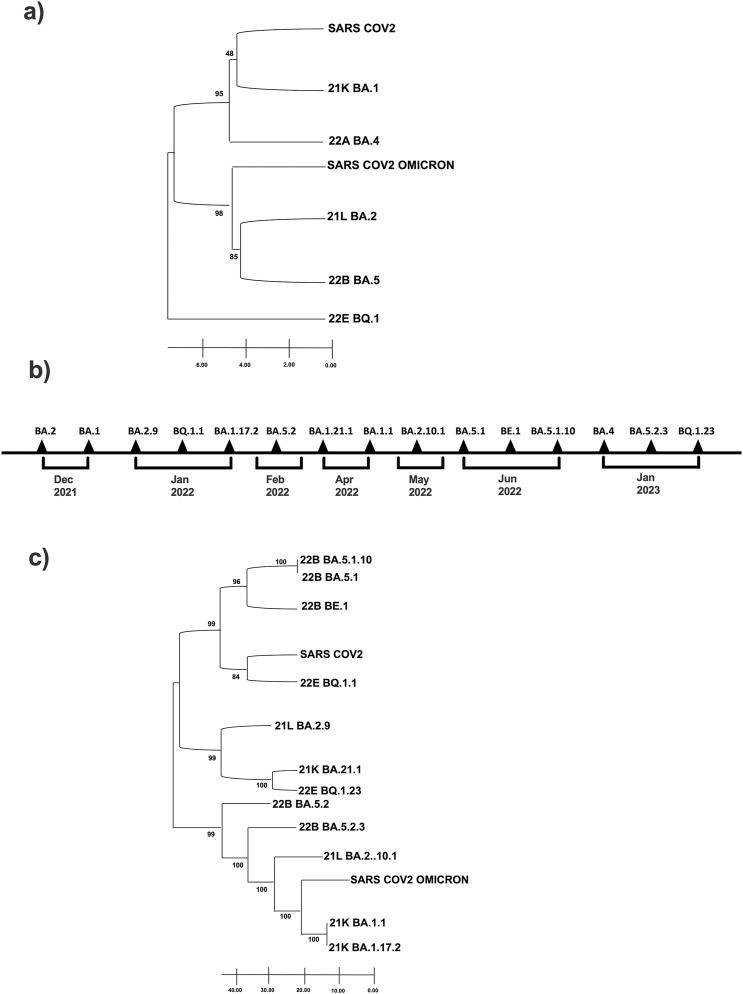
Phylogenetic tree of Omicron clade and variants identified in our 38-sample collection. **(A)** Clade and **(C)** Variants are related to first historically known sequences, SARS-CoV-2 and SARS-CoV-2 Omicron. Evolutionary analyzes were conducted using MEGA11; **(B)** Timeline of Sars-CoV-2 infection variants in the time.

To uncover the evolutionary relationships between the five Omicron clades identified with the original Omicron variant (B.1.1.529) and the first known SARS-CoV-2 sequence, we performed a phylogenetic analysis using the UPGMA method and a bootstrap test (Figure 2).

The phylogenetic tree reveals that clade 21K is evolutionarily closest to the ancestral SARS-CoV-2, while clades 21L and 22B exhibit the most substantial similarity to the Omicron lineage (Figure 2A). Most notably, clade 22E is the most divergent and follows an entirely separate evolutionary path: it is positioned as the most evolutionarily distant from SARS-CoV-2 and Omicron (Figure 2A). Also, we performed a temporal onset description of the SARS-CoV-2 variant’s appearance (Figure 2B), and we compared them with the sequence data of the virus found in Italy during the same period reports provided by the Istituto Superiore di Sanità (ISS) (see Supplementary Table S3).

Finally, we have established the phylogenetic relationships among the 15 subvariants distributed within the five variants. A second phylogenetic analysis was created using the UPMGA method and bootstrap test to understand the evolutionary distance of the variants from the first SARS-CoV-2 and the first Omicron. The variant BQ.1.1 of clade 22E is evolutionarily the closest subvariant of the first SARS-CoV-2. Therefore, looking at such a phylogenetic tree, it appears that the two subvariants evolutionarily closest to Omicron and at the same time farthest from SARS-CoV2 seem to be BA.1.1 and BA.1.17.2 both 21K clade. In contrast, the farthest from Omicron are precisely BA.5.1, BA.5.1.10, and BE.1, all from the 22B clade (Figure 2C).

### 3.3 Mutational dimension

**FIGURE 3:**
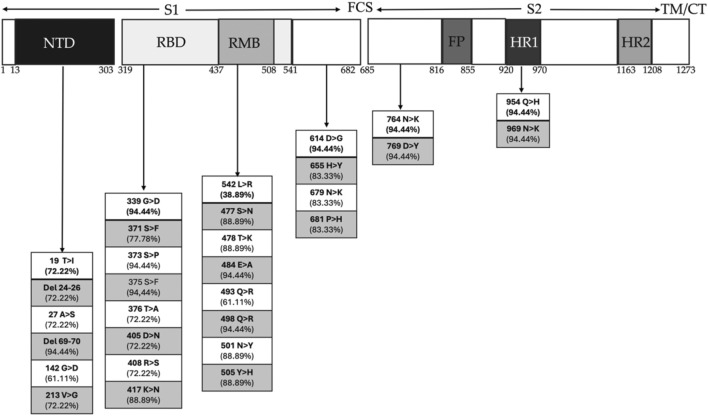
Schematic representation of Spike genome sequence of SARS-CoV-2 with domain organization and amino acid changes. The S1 subunit of the Spike protein is composed of the N-terminal domain (NTD) and the receptor binding domain (RBD), within which there is the receptor motif binding (RMB), which directly contacts ACE2 and the C-terminal domain (CTD). The S2 subunit is composed of fusion peptide (FP) subdomains, two heptad repeats (HR1 and HR2), a transmembrane helix, and a cytoplasmic tail (TM/CT). The furin cleavage site (FCS) separates the two subunits. Mutations are present in percentages in each of the regions of the Spike protein for the most severe variants.

Our analysis revealed 71 mutations, accounting for more than 25%. Out of all amino acid mutations detected, 56 led to substitutions and 15 to deletions. Detailed sequence analysis identified a comprehensive list of these mutations across each SARS-CoV-2 genomic region within our patient’s cohort (Supplementary Table S4). While most of the observed mutations were typical of Omicron, several others diverged from those present in the initial Omicron B.1.1.529 strain. Notably, the variant BQ.1.1 from clade 22E exhibited the highest number of amino acid mutations in the spike protein, while BA.1 from clade 21K showed the fewest (Supplementary Table S4). We specifically focused on amino acid mutations in the genomic coding region of the spike protein, the region with the highest mutation rate and a crucial target for vaccine design. As expected, the S gene exhibited the largest mutation number (n = 38) (Supplementary Table S4) (Figure 3). Our findings highlight multiple recurrent mutations: 6 aminoacidic mutations in the N-terminal domain (NTD) of the S1 subunit, 8 in the receptor-binding domain (RBD), 8 in the receptor-binding motif (RBM), 6 surrounding the furin cleavage site (FCS), and 2 additional mutations (Figure 3). Patients infected by BA.2, BA.5.1 and BA5.2 variants showed severe symptomatology at the history stage: all had five aminoacidic deletions, all of them within the N-terminal chain S1 (33–373): deletions of histidine and valine at positions 69 and 70, respectively (H69-, S: V70-), common to the Omicron variant B.1.1.529; deletions of the nonpolar amino acids leucine and proline at positions 24 to 26 (L24-, P25-, P26-), typical of these subvariants.

Then, they exhibit all of 22 common substitutions shared with Omicron, including 12 within the RBD region of the S1 subunit (G339D, S371F, S373P, S375F, K417N, N440K, S477N, T478K, E484A, Q498R, N501Y, Y505H), 1 within the S1/S2 proteolytic cleavage region (P681H), and 4 within the S2 region (N764K, D796Y, Q954H, N969K).

### 3.4 Long-term COVID-19 symptoms

**FIGURE 4:**
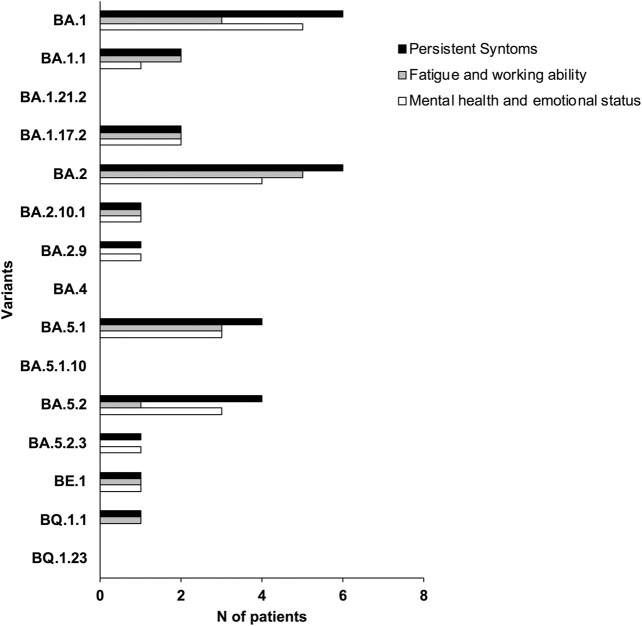
Symptom prevalence estimates in our sample collection. Each bar represents the per-centage of respondents who experienced that symptom related with the specific variant.

To investigate the long-term effects of COVID-19 after 1–3 years, questionnaires were administered to all 38 participants. The symptoms of individual patients were subsequently associated with Omicron variants (Figure 4). Our data reveal that patients infected by BA.1.1, BA.17.2, BA.2.10.1, BA.5.1 and BE.1 variants experienced persistent symptoms even after 1–3 years, negatively impacting workability, mental health and emotional state (Figure 4). Although based on a limited sample, our data suggest that the long-term effects of COVID-19 are especially marked in patients over 50, except BA.2.10. Further research is essential to validate the observed link between age, specific variants, and persistent COVID-19 symptoms (Table 3).

**TABLE 3:** Distribution of SARS-CoV-2 Pango lineages variants respect to the age of patients.

Variant	Total N of Patients	Age (Mean ± SD)
BA.1	6	56.6 (±17.3)
BA.1.1	2	53.8 (±18.3)
BA.1.17.2	3	48.0 (±16.2)
BA.1.21.1	1	22.0 (−)
BA.2	7	48.6 (±16.5)
BA.2.10.1	1	37.0 (−)
BA.2.9	1	67.0 (−)
BA.4	1	52.0 (−)
BA.5.1	5	56.4 (±13.2)
BA.5.1.10	1	37.0 (−)
BA.5.2	4	49.5 (±14.3)
BA.5.2.3	1	49.0 (−)
BE.1	1	53.0 (−)
BQ.1.1	3	44.3 (±20.2)
BQ.1.23	1	50.0 (−)

## 4 Discussion

RNA viruses, such as Coronaviruses, constantly evolve through mutations in their genome. Mutations of the SARS-CoV-2 virus have been observed worldwide since the beginning of the pandemic. Given the rapidity with which these mutations occur, the scientific community is now more critical than ever to understand the potential mechanisms by which these alterations are positively selected along the evolution of SARS-CoV-2 (Magazine et al., 2022). Therefore, comprehensive genomic profiling using NGS has identified clinically actionable alterations in patients with different variants that can support the selection of suitable therapy (Pei et al., 2023).

In this scenario, in our prospective pilot study, 38 SARS-CoV-2-positive patients were collected and stratified by age, sex, vaccination, symptomatic status, and place of residence. In our sample collection, the major presence of SARS-CoV-2 infection was observed in the 30–40 age range. Of this group, more than half showed severe symptoms during infection.

First of all, sequencing data showed the dominant presence in the sample collection of 5 Omicron clades: 22A, 22B, 22E, 21K and 21L; the most abundant of these clades were 21K and 22B with a frequency of 30%. Belonging to these 5 clades we can find their respective 15 variants: BA1, BA1.1, BA1.17.2, BA1.21.1, BA2, BA2.9, BA2.10.1, BA4, BA5.1, BA5.1.10, BA5.2, BA5.2.3, BE.1, BQ1.23, BQ1.1 identified. On the other hand, the data obtained indicate that the most abundant variant from December 2021 to February 2023 is BA.1 for the municipality of Bacoli; while for the municipality of Monte di Procida is BA.2, and for Pozzuoli is BA.5.1. Our data are in accordance with the SARS-CoV-2 variants distribution reported in the Istituto Superiore di Sanità (Report No. 26 of Dec. 2, 2022), in which it is shown that the Omicron variant accounts for almost all (99.96%) of the deposited sequencing and that the BA.5 variant is predominant (92.41%), with 142 different lineages identified, including the parental lineage (Stefanelli et al., 2022).

Interestingly, the three urban communities we analyzed are relatively independent. The genetic evolution of SARS-CoV-2 has occurred in a continuous adaptation to new human hosts. Being an RNA virus, SARS-CoV-2 has a high rate of mutation and recombination events due to low RNA polymerase fidelity. The virus variants appear to spread more effectively in susceptible hosts than in the initial epidemic virus and may also be more resistant to naturally acquired or vaccine-induced immunity (Cai et al., 2021). This phenomenon shows us how rapid the virus’ ability to mutate is in space and time, given three communities that are very close geographically and temporally. All variants affect the male sample collection except for variants BQ.1.23 and BA.1.21.1, which affect only females. Our data are in line with what is reported in the literature, which reveals that men are more prone to getting ill than women (Rodrigues et al., 2024; Windi et al., 2023). Secondly, we observed the variations of the Spike protein for each of the 15 variants to reconstruct best the evolutionary relationships that emerged from the phylogenetic analysis. The 21K clade is the one evolutionarily closest to the first SARS-CoV-2. From the node joining 21K and SARS-CoV-2 originates the branch of 22A, which developed at the same evolutionary time as the first Omicron (B.1.1.529), albeit independently of it. The clades 21L and 22B are the closest to Omicron, while 22E is the most distant variant from SARS-CoV-2 and Omicron (B.1.1.529); the latter moves entirely independently of both placed clades. The bootstrap values obtained are high; therefore, the tree’s significance is confirmed (Felsenstein, 1985). A second phylogenetic tree was created using the UPMGA method and bootstrap tests to understand the evolutionary distance of the variants from the first Omicron (B.1.1.529) and the first SARS-CoV-2. This analysis showed variants BA.5.1 and BA.5.1.10 of clade 22B and BA.1.1 and BA.1.17.2 of clade 21K are temporally older and distant from both references chosen. These evolutionary changes were most likely selected during adaptation of SARS-CoV-2 to our population (Ronsard et al., 2014; Ronsard et al., 2015).

Variant BE.1 of clade 22B originated from BA.5.1 and BA.5.1.10 proceeds independently. From the same node from which it originates BE.1, SARS-CoV-2 and BQ.1.1 of clade 22E also branch off. These three variants are located at the same distance. From the branch leading to SARS-CoV-2, the branch leading to variant BA.2.9, BA.1.21.1 and BQ.123 originates. Going back evolutionarily, we then find BA.5 from which cascade BA.5.2.3, BA.2.10., the first Omicron B.1.1.259, BA.1.1 and BA.1.17.2. The phylogenetic analysis highlighted a great intrinsic variability of Omicron, which as the literature suggests has more than 50 mutations compared to the wild strain (Sun et al., 2022); this variability is simultaneously synonymous with its infectious capabilities. Our emerging data from the sequencing of viral variants present in the territory of the Phlegraean area of Campania are consistent with the prevalence data observed in the national monitoring reports provided by the ISS (Istituto Superiore di Sanità, 2024). As highlighted by national data, we also found a territorial prevalence of the Omicron variants BA.1 and BA.2 and their respective subvariants in subjects analyzed between December and May 2022, while the Omicron variants BA.4 and BA.5 and their respective subvariants were detected later between June and July 2022 (Rachiglio et al., 2021).

Yanhua Li and collaborators have highlighted that the BA.2 variant causes long-term effects on both the metabolism and the immune system (Li et al., 2024). In addition, we showed amino acid substitutions and deletions for each portion of the viral genome. Special attention was paid to the variability found for the portion encoding the Spike protein for each of the 15 variants identified.

Inside the NTD, we find deletions of histidine and valine at positions 69 and 70, respectively, which are also expected to Omicron variant B.1.1.529 (Souza et al., 2022), but also deletions of the nonpolar amino acids leucine and proline at positions 24 to 26, which are typical of the variants we found with more severe symptoms (BA.1.1, BA.1.17.2, BA.2, BA.5.1, BA.5.2.3) (Souza et al., 2022).

Among the common substitutions within Omicron genome, 12 falls within the RBD and RMB region of the S1 subunit (G339D, S371F, S373P, S375F, K417N, N440K, S477N, T478K, E484A, Q498R, N501Y, Y505H), one falls within the S1/S2 proteolytic cleavage region (P681H), and four falls within the region that makes up S2 (N764K, D796Y, Q954H, N969K).

Prominent among these substitutions common to all the more severe variants and retained by the Omicron variant are the 417-position substitution of lysine for asparagine (K417N), the 501-position substitution of asparagine for tyrosine (N501Y), and the 484-position substitution of glutamic acid for lysine (E484K); these are known to improve the binding efficiency for the human ACE2 receptor (Leung et al., 2021; Tegally et al., 2021). These mutations are located in the spike protein’s receptor binding domain (RBD). They can alter the structure of the spike protein, which is the primary target of vaccine-induced neutralizing antibodies. This could significantly impact the efficacy of vaccines designed based on the original viral strain, and they may increase the likelihood of disruptive infections even in vaccinated individuals (Tian et al., 2021). The presence of these mutations in the circulating variants (such as the Beta, Gamma and Omicron variants) has prompted vaccine manufacturers to consider updating or modifying their vaccines to suit these variants better. Moreover, recalls targeting these specific mutations may be necessary to maintain vaccine efficacy over time (Li et al., 2022).

Interestingly, the substitutions of 655-histidine with tyrosine (H655Y), 679-asparagine with lysine (N679K) and 796-aspartic acid with tyrosine (D796Y) were found. It was hypothesized that their proximity to the furin cleavage site in the spike protein is associated with increased infectivity (Gong et al., 2021). Other Omicron-conserved and common substitutions are the substitutions of the polar uncharged amino acids serine at positions 371, 373, and 375 with the nonpolar amino acids leucine, proline, and phenylalanine, respectively (S371L, S373P, S375F). These substitutions provide increased hydrophobicity that stabilizes the RDB region and promotes evasion of antibody binding (Zhao et al., 2022).

The recent literature suggests that combining the new mutations in Omicron led the variant to have higher infectivity than the original Wuhan-Hu-1 and Delta variants. However, the severity is believed to be lower due to reduced syncytial formation and less multiplication in human lung tissue. Perhaps most challenging is that several studies indicate that the efficacy of available vaccines has been reduced against the Omicron variant (8–127-fold reduction) compared with the Wuhan-Hu-1 variant. In contrast, administration of the booster vaccine compensates for the reduction, improves efficacy 12–35-fold, and confers protection from developing severe disease in the infection. It is known that Omicron BA.4 and BA.5 variants, with attached related subvariants, are reported to be more transmissible and resistant to immunity generated by earlier variants, such as Omicron BA.1, or by most monoclonal antibodies (Shrestha et al., 2022).

Several studies have shown that the Omicron variant has significantly greater immune evasion properties than its predecessor. Omicron’s immune evasion ability has contributed to its rapid global spread, and universal booster vaccines can restore Omicron’s protection to some extent (Ailan et al., 2022). These data agree with the findings of our territorial analysis of vaccinated patients in which only the Omicron variant and its subvariants are present in the territory.

Finally, we collected data on Long-term COVID-19 in our sample collection. Long-term COVID-19 is a multisystem condition that often includes severe symptoms following severe acute respiratory syndrome (SARS-CoV-2) infection. At least 65 million individuals worldwide are affected by long-term COVID-19, based on a conservative estimate of the incidence of 10% of infected people and more than 651 million documented cases of COVID-19 worldwide. The number of COVID-19 cases is likely higher than the documented 651 million due to many unreported or undiagnosed cases. Long-term COVID-19 is associated with all ages and all severities of acute phase disease, with the highest percentage of diagnoses between the ages of 36 and 50 (Lara Bull-Otterson et al., 2022; Ceban et al., 2022).

In our study, we ultimately observed that patients infected by BA.2.10.1 and BE.1 variants presented effects of Long-term COVID-19 with persistent symptoms: fatigue, problems with workability, and mental health and emotional state. Interestingly, BE.1 evolved evolutionarily closer to SARS-CoV-2, while BA.2.10.1 was phylogenetically closer to Omicron.

Long-term COVID-19 includes a range of ongoing health issues, such as fatigue, cognitive impairment, and respiratory problems that persist long after the initial infection. The BA.2.10.1 and BE.1 subvariants could exacerbate these symptoms due to their ability to evade immunity, thereby increasing the likelihood of reinfection and prolonging the body’s recovery process. Moreover, studies have shown that even mild cases of COVID-19 can result in significant long-term immune system alterations, which might explain some of the persistent symptoms observed in Long-term COVID-19 (Yang et al., 2022; Varghese et al., 2023).

Furthermore, the emergence of these Omicron subvariants could necessitate re-evaluating our current therapeutic and vaccination strategies. The effectiveness of existing vaccines, particularly those designed for earlier strains, may be diminished against these new variants. This underscores the need for updated vaccines or booster shots targeting Omicron variants.

In agreement with data in the literature, patients in our study who reported presenting with all the symptoms of long-term COVID-19 were over 50 years old, considering the limitations of our research.

## 5 Conclusion and study limitations

Our pilot study highlighted which variants were associated with long-term COVID-19 effects from 1–3 years after infection. So, thanks to NGS method, we could finally observe that some variants SARS-CoV-2 were associated with consistent effects on the severity and persistence of post-COVID sequelae, but currently, the number of patients analyzed is too low for a significant conclusion. In the future, the genomic approach supported by bioinformatics analyses could represent the gold standard for viral infections for differential diagnosis and personalized therapy. On the other hand, several limitations should be considered: participants were recruited from a single testing analysis center. While this allows us to capture participants’ experience of their sequelae, it also introduces the risk of misclassification, as perceptions may evolve, and some participants’ symptoms could be due to a health condition unrelated to COVID-19. Moreover, some variants were underrepresented. Finally, a larger group of patients for an epidemiological study of variants over time and in space would have been useful.

## Data Availability

The original contributions presented in the study are publicly available. This data can be found here: GISAID repository, https://gisaid.org. Accession IDs are available in Supplementary Table S1.
