# Supplementary material for: Pilot study: a descriptive-retrospective analysis of SARS-CoV-2 variants distribution and phylogenesis in the Phlegraean area
Source: Front Mol Biosci. 2025 Feb 27;12:1536953. doi: 10.3389/fmolb.2025.1536953 (PMC11903270; doi:10.3389/fmolb.2025.1536953)
Supplement: Supplementary file 2 [file Table3.docx]

**Table S3.** Percentage of SARS-CoV-2 Pango lineages Variants distribution in Italy.

| **Data** | **Variant** | **Distribution in Italy** |
| --- | --- | --- |
| **December 2021 February 2022** | BA.1  BA.2 | 90**%** |
| **March 2022** | BA.1.1  BA.2  BA.3 | 31.05**%**  30**%**  0.02**%** |
| **April 2022** | BA.2  BA.2.9  BA.1.1 | 62.8**%**  12.9**%**  9.5**%** |
| **May 2022** | BA.2 | 93% |
| **June 2022** | BA.2  BA.4  BA.5 | 62.8%  23.15%  23.15% |
| **July 2022** | BA.5 | 81.7% |
| **August 2022** | BA.5 | 90.08 |
| **September 2022** | BA.5  BA.4 | 93.8%  6.2% |
| **October 2022** | BA.5  BA.4 | 90% |
| **November 2022** | BA.5  BQ.1.1  BF.7  BA.5.2  BA.5.1 | 92.41%  13.25%  12.32  11.69%  8.94 |
| **December 2022** | BA.5  BQ.1.1 | 30.84% |
| **January 2023** | BA.5  BQ.1.1  BA.2  BAXBB.1.5 | 86.3%  38.9%  9.8%  9.8% |
