# Supplementary material for: Pilot study: a descriptive-retrospective analysis of SARS-CoV-2 variants distribution and phylogenesis in the Phlegraean area
Source: Front Mol Biosci. 2025 Feb 27;12:1536953. doi: 10.3389/fmolb.2025.1536953 (PMC11903270; doi:10.3389/fmolb.2025.1536953)
Supplement: Supplementary file 4 [file Table4.docx]

**Table S4.** The amino acid mutations corresponding to SARS-CoV-2 genes, found in each viral genome region.

| **Genome region** | **AA position and change** | **Number of patients** |
| --- | --- | --- |
| ORF1 a | S135R | 27 |
|  | K856R | 10 |
|  | L2084I | 10 |
|  | T3255I | 37 |
|  | P3395H | 37 |
|  | S3675- | 38 |
|  | G3676- | 38 |
|  | F3677- | 27 |
|  | L3674- | 11 |
|  | I3758V | 10 |
|  | G1307S | 26 |
|  | L3027F | 23 |
|  | T3090I | 27 |
| ORF1 b | P314L | 33 |
|  | I566V | 37 |
|  | R1315C | 26 |
|  | T2163I | 25 |
| 0RF 3a | T223I | 26 |
| ORF9b | P10S | 35 |
|  | E27- | 37 |
|  | A29- | 37 |
| E gene | T9I | 37 |
| M gene | D3G | 11 |
|  | D3N | 15 |
|  | Q19E | 31 |
|  | A63T | 38 |
| N gene | P13L | 38 |
|  | E31- | 37 |
|  | R32- | 37 |
|  | S33- | 37 |
|  | R203K | 37 |
|  | G204R | 38 |
|  | S413R | 27 |
| S gene | T19I | 26 |
|  | L24- | 26 |
|  | P25- | 26 |
|  | P26- | 26 |
|  | A27S | 26 |
|  | A67V | 10 |
|  | H69- | 30 |
|  | V70- | 30 |
|  | T95I | 11 |
|  | G142D | 23 |
|  | Y144- | 12 |
|  | V213G | 26 |
|  | G339D | 31 |
|  | S371F | 26 |
|  | S373P | 32 |
|  | S375F | 32 |
|  | T376A | 26 |
|  | D405N | 26 |
|  | R408S | 26 |
|  | N440K | 15 |
|  | L452R | 17 |
|  | S477N | 30 |
|  | T478K | 30 |
|  | E484A | 30 |
|  | F486V | 17 |
|  | Q493R | 13 |
|  | Q498R | 30 |
|  | N501Y | 30 |
|  | Y505H | 29 |
|  | T547K | 11 |
|  | H655Y | 35 |
|  | N679K | 35 |
|  | P681H | 35 |
|  | N764K | 37 |
|  | D796Y | 37 |
|  | N856K | 11 |
|  | Q954H | 36 |
|  | N969K | 37 |
|  | L981F | 11 |
